# The Development of a Rapid, Cost-Effective, and Green Analytical Method for Mercury Speciation

**DOI:** 10.3390/toxics12060424

**Published:** 2024-06-11

**Authors:** Patrícia Cristina Costa Ladeira, Caroline Cristine Augusto, Bruno Alves Rocha, Jairo Lisboa Rodrigues, Giovanna de Fátima Moreno Aguiar, Bruno Lemos Batista

**Affiliations:** 1 Center for Natural and Human Sciences (CCNH), Federal University of ABC (UFABC), Santo André 09210-580, SP, Brazil; patricia.ladeira@yahoo.com.br (P.C.C.L.); caroline.augusto@ufabc.edu.br (C.C.A.); 2 Department of Clinical Analyses, Toxicology and Food Sciences, School of Pharmaceutical Sciences of Ribeirao Preto, University of Sao Paulo, Avenida do Cafe s/nº, Ribeirão Preto 14040-903, SP, Brazil; farmbrunorocha@gmail.com; 3 Institute of Chemistry, Federal University of Alfenas, Alfenas 37130-001, MG, Brazil; 4 Instituto de Ciência, Engenharia e Tecnologia, Universidade Federal dos Vales do Jequitinhonha e Mucuri, Teófilo Otoni 39803-371, MG, Brazil; jairo.rodrigues@ufvjm.edu.br; 5 Agência USP de Inovação, University of São Paulo, Avenida Bandeirantes 3900, Ribeirão Preto 14040-900, SP, Brazil; aguiar.giovanna@outlook.com

**Keywords:** mercury speciation, fast method, UHPLC-ICP-MS, ICP-MS, environmental monitoring

## Abstract

Mercury is a naturally occurring metal found in various inorganic and organic forms within the environment. Due to its high toxicity, there is global concern regarding human exposure to this element. The combination of high-performance liquid chromatography and inductively coupled plasma mass spectrometry (HPLC-ICP-MS) is commonly used to analyze the different forms of mercury in a sample due to its high sensitivity and ability to selectively detect mercury. However, the traditional HPLC-ICP-MS methods are often criticized for their lengthy analysis times. In this study, we have refined the conventional approach by transitioning to ultra-high performance liquid chromatography coupled with inductively coupled plasma mass spectrometry (UHPLC-ICP-MS). This modification has resulted in significant reductions in runtime as well as reagent and argon usage, thereby offering a more rapid, environmentally friendly, and cost-effective method. We successfully adapted an HPLC-ICP-MS method to UHPLC-ICP-MS, achieving the analysis of Hg^2+^ and MeHg^+^ within 1 min with a mobile phase consumption of only 0.5 mL and a sample volume of 5.0 µL; this is a major advance compared to HPLC analysis with run times generally between 5 and 10 min. The method’s performance was assessed by analyzing muscle and liver tissue samples (serving as reference material) from fish, demonstrating the versatility of the method in relation to different complex matrices.

## 1. Introduction

The pervasive presence of mercury in the environment and its various species pose significant health risks due to their high toxicity, which is linked to a range of diseases. Mercury’s natural occurrence and widespread use in numerous applications lead to its environmental distribution, allowing for its absorption by flora and fauna and subsequent pathways of human exposure [1,2,3,4].

While HPLC has been traditionally employed in chemical speciation analysis, often coupled with atomic absorption [5] and atomic fluorescence [6,7], ICP-MS has become a preferred methodology. This preference is due to its seamless integration, which allows for direct interfacing between the chromatographic output and the nebulizer inlet, and its compatibility with the flow rates of both systems, along with its superior sensitivity and selectivity [8].

HPLC-ICP-MS has been increasingly used to analyze the different forms of mercury in various substances for multiple purposes. For instance, the assessment of human mercury exposure has been conducted through the analysis of blood and hair, as demonstrated by Petry-Podgórska et al. [9]. Similarly, Zhu et al. employed magnetic solid-phase extraction (SPE) to quantify Hg^2+^, and organic species of mercury in fish and water samples, which are critical for evaluating environmental and food contamination [10]. Zhou et al. reported the use of magnetic SPE for analyzing water and fish samples [11]. These studies typically achieve the determination of Hg^2+^ and MeHg^+^ within approximately 4 min. However, when additional species such as ethylmercury (EtHg^+^) and PhHg^+^ are present, the analysis duration can extend to around 10 min.

The transition from HPLC to ultra-high-performance liquid chromatography (UHPLC) represents a significant advancement, offering more rapid analyses, which is beneficial for increasing analytical throughput and reducing reagent consumption—key considerations in Green Chemistry [12]. The evolution of UHPLC is marked by the development of sub-2 µm stationary phase particles and instruments capable of withstanding high pressures (up to 100 MPa), which together enhance chromatographic performance [13]. The reduced particle size facilitates a quicker equilibrium between the stationary and mobile phases due to shallower diffusion paths, thus shortening retention times. Additionally, the use of more compact columns yields a higher number of theoretical plates per unit length, maintaining efficiency even in shorter columns. Moreover, the smaller internal diameter of these columns allows for a higher linear velocity of the mobile phase, further accelerating the elution of species [13]. Therefore, the purpose of this investigation was to modify a preexisting HPLC-ICP-MS technique for mercury speciation to a UHPLC-ICP-MS procedure, thereby attaining faster analysis times, and contributing to reducing the use of solvents and the consequent analytical cost through the use of the UHPLC technique, which is not yet reported in any study published in this field of application.

## 2. Materials and Methods

### 2.1. Materials and Reagents

The chemical reagents (analytical grade) were utilized as received, except for hydrochloric acid (HCl, 36% *m/v*), which was distilled using a PFA sub-boiling distillation system (Savillex DST-1000 Acid Purification System, Eden Prairie, MN, USA). All aqueous solutions were prepared with water purified to a resistivity of 18.2 MΩ·cm, obtained from a PURELAB^®^ Option Q system (Elga, Cotia, Brazil). For the calibration curve, a 10 mg L^−1^ inorganic Hg standard solution (PerkinElmer, Shelton, CT, USA) and a 1000 mg L^−1^ methylmercury (MeHg^+^) solution were employed. The latter was prepared by dissolving MeHgCl (Sigma Aldrich, St. Louis, MO, USA) in an aqueous medium in the presence of nitric acid 1.0% (*v/v*) and methanol 5.0% (*v/v*). Hydrochloric acid, L-cysteine, and 2-mercaptoethanol were obtained from Sigma-Aldrich (St. Louis, MO, USA). A mixture of methanol (Sigma Aldrich, St. Louis, MO, USA) and an aqueous solution containing ammonium acetate (Merck, Darmstadt, Germany), L-cysteine, and 2-mercaptoethanol was used as the mobile phase. 

### 2.2. Instrumental Apparatus and Analysis Conditions

The speciation studies were conducted with an Agilent 1290 Infinity II Liquid Chromatography System (Agilent, Waldbronn, Germany) coupled with an Agilent 7900 Inductively Coupled Plasma Mass Spectrometer (Thermo Scientific, Hachioji, Japan). A stainless-steel capillary with an inner diameter of 0.17 mm was used to connect the output of the chromatographic column to the ICP-MS nebulizer inlet. Two Agilent ZORBAX Eclipse Plus C18 columns were assessed for the stationary phase. One column, measuring 4.5 mm × 150 mm with a 3.5 µm particle size, was used for the HPLC. The second one, measuring 2.1 mm × 50 mm with a 1.8 µm particle size, was used for the UHPLC. 

Before assessing the UHPLC-ICP-MS system, we examined the method proposed by Rodrigues et al. [14]. The authors used a C18 column (4.5 mm × 150 mm, 3.5 µm), a stationary phase similar to the HPLC column applied in the proposed method. The mobile phase consisted of a 95% aqueous solution containing 2-mercaptoethanol (0.05%), L-cysteine (0.40%), and ammonium acetate (0.06 mol L^−1^), supplemented with 5% methanol, flowing at a rate of 500 µL min^−1^ [14].

Standard solutions were prepared in an aqueous diluent containing HCl (0.10%), L-cysteine (0.05%), and 2-mercaptoethanol (0.10%). To determine the retention times of Hg^2+^ and MeHg^+^, 20 µL of 5.0 µg L^−1^ solutions were individually injected, followed by a combined solution of both species at the same concentration. The acquisition time was set to 15 min.

After HPLC testing, the method was adapted for UHPLC using a C18 column (2.1 mm × 50 mm; 1.8 µm). The volume of injection was reduced to 5.0 µL, and the acquisition time was shortened to 1.5 min. All other parameters remained as per the initial study. Chromatograms of each species were first obtained individually to confirm retention times, followed by an analysis of a mixed solution. The analytical calibration curve ranging from 1 to 10 µg L^−1^ was prepared from stock solution. Table 1 summarizes the ICP-MS instrumental parameters used for speciation.

### 2.3. Species Stability

The sample preparation process entailed subjecting the sample to sonication using an ultrasonic bath (Eco-Sonics, Ultronique, Indaiatuba, Brazil). In order to evaluate the possibility of species interconversion and volatilization during this procedure, a triplicate of standard aqueous solutions (concentrations of 1.0, 5.0, and 10.0 µg L^−1^) was exposed to sonication for 15 and 30 min in the diluent media. The stability of the solutions was monitored by storing them in amber vials at room temperature (~25 °C) and comparing chromatograms from freshly prepared solutions with those stored for one week. Additionally, the stability of solutions kept in amber vials was compared to that of solutions stored in polypropylene tubes over a 72 h period.

### 2.4. Sample Preparation for Total Hg and Chemical Speciation

For total Hg, 100 mg of lyophilized samples (muscle and liver) of *Bonito* fish (species *Katsuwonus pelamis*), collected in 2018 in the Brazilian northeast coast, was digested with 1.5 mL of aqua regia (3 parts of sub-boiled HCl and 1 part of sub-boiled HNO_3_) in an ultrasound bath for 30 min. The volume of the samples was made up to 30 mL with an aqueous solution containing 2-mercaptoethanol 0.05% *v/v*.

For chemical speciation, we followed the procedure described by Batista et al.^15^ For this, we precisely measured and deposited 100 mg of fish muscle or liver samples into 15 mL polypropylene conical tubes. Around 10 mL of an extraction solution, consisting of HCl (0.10%), L-cysteine (0.05%), and 2-mercaptoethanol (0.10%), was added to these samples. The samples were agitated by hand to ensure uniformity and then sonicated for 30 min in an ultrasonic bath (Eco-Sonics, Ultronique, Indaiatuba, Brazil) to enhance the extraction of different forms of mercury.^15^ After sonication, it was centrifuged at a speed of 3500 rotations per min for 5 min (SL-700 centrifuge, Solab, Piracicaba, Brazil). Prior to analysis, the resultant supernatant was filtered using a 0.20 µm syringe filter (Sartorius, Göttingen, Germany) to eliminate any particulate matter. Once there were no specific concentrations of Hg chemical species for the samples, the concentrations of the individual species were summed and the values compared to the total concentration of Hg previously found.

## 3. Results and Discussion

**Figure 1:**
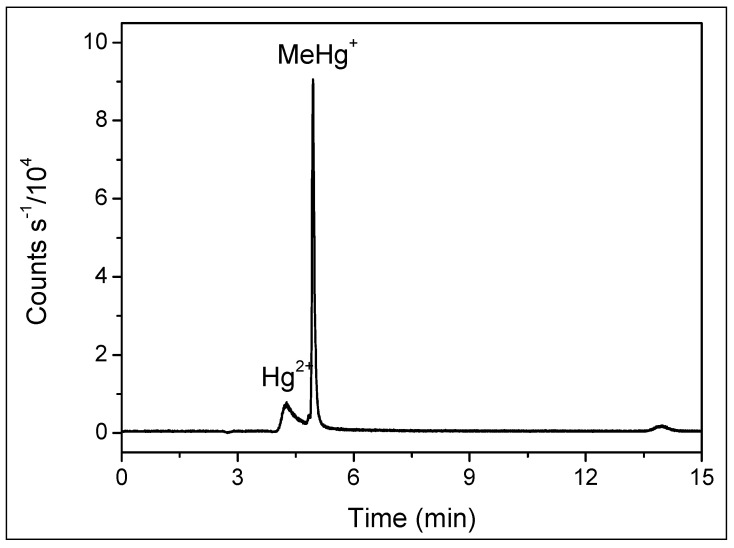
Chromatogram obtained by HPLC-ICP-MS for 20 µL of solution containing Hg^2+^ and MeHg^+^ 5.0 µg L^−1^. Mobile phase: 0.05% *v/v* of 2-mercaptoethanol, 0.40% *m/v* of L-cysteine, 0.06 mol L^−1^ of ammonium acetate, and 5.0% *v/v* of methanol at 0.5 mL min^−1^. Stationary phase: column C18 4.6 mm × 150 mm, particle diameter 3.5 µm.

**Figure 2:**
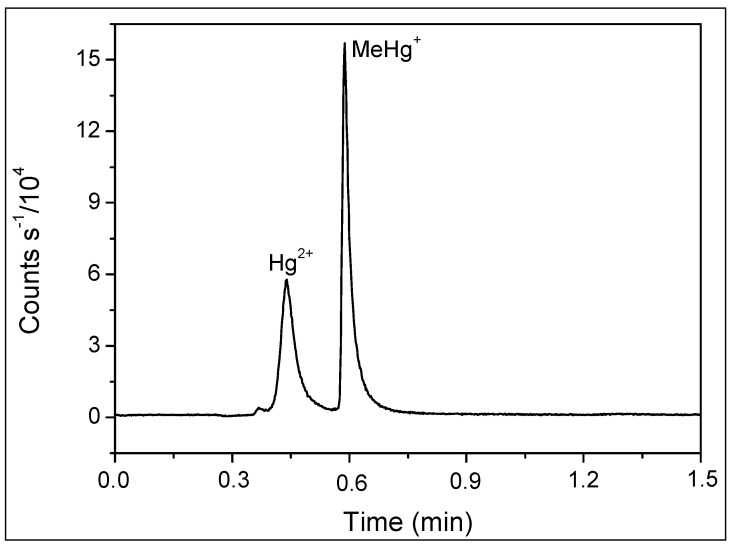
Chromatogram obtained by UHPLC-ICP-MS for 5 µL of solution containing Hg^2+^ and MeHg+ 5.0 µg L^−1^. Mobile phase: 0.05% *v/v* of 2-mercaptoethanol, 0.40% *m/v* of L-cysteine, 0.06 mol L^−1^ of ammonium acetate, and 5.0% *v/v* of methanol at 0.5 mL min^−1^. Stationary phase: column C18 4.6 mm × 150 mm, particle diameter 1.8 µm.

Using a C18 column in reverse-phase chromatography with an aqueous mobile phase, our HPLC-ICP-MS analysis resulted in retention times of 4.3 min for inorganic mercury and 4.9 min for methylmercury, as shown in Figure 1. Despite the method’s effectiveness in mercury speciation, the extended duration of chromatographic runs increased both the analytical costs and the consumption of reagents and argon. Transitioning to UHPLC, we observed that retention times decreased to 0.4 and 0.6 min for Hg^2+^ and MeHg^+^, respectively, without compromising separation efficiency (Figure 2). This modification resulted in an approximately tenfold reduction in retention time and a 90% reduction in mobile phase consumption, significantly enhancing both the resolution and sensitivity of the peaks.

The improved UHPLC-ICP-MS method aligns with the literature on the benefits of UHPLC for maintaining separation efficiency while reducing analysis time and reagent use [13]. Although direct comparisons of HPLC to UHPLC for mercury speciation are absent in the current literature, analogous conversions for organic compounds support our findings [15,16,17]. The results demonstrate that the development method is a reliable and successful strategy for determining the different forms of mercury, with notable enhancements in efficiency and cost-effectiveness. The method’s high sensitivity and selectivity (slopes of 943 and 1287, R2 of 0.9991 and 0.9994, and limits of detection (LoD, in µg L^−1^) of 0.27 and 0.07 for Hg^2+^ and MeHg^+^, respectively) could enhance the efficiency of environmental monitoring and risk assessment studies for mercury contamination, allowing for increased throughput. In comparison to the other literature, the proposed methodology showed shorter retention times (approximately 0.4 and 0.6 min for inorganic mercury and methylmercury, respectively) compared to 0.6 and 1.0 min found by Batista et al. [18]. Also, the limits of detection for both species were comparable to Batista et al. and Rodrigues et al. [14,18], who found 0.25 and 0.1 µg L^−1^ for Hg^2+^ and MeHg^+^, respectively, and lower than 0.8 and 0.7 µg L^−1^, which were the results found by Santoyo et al. [19].

**Figure 3:**
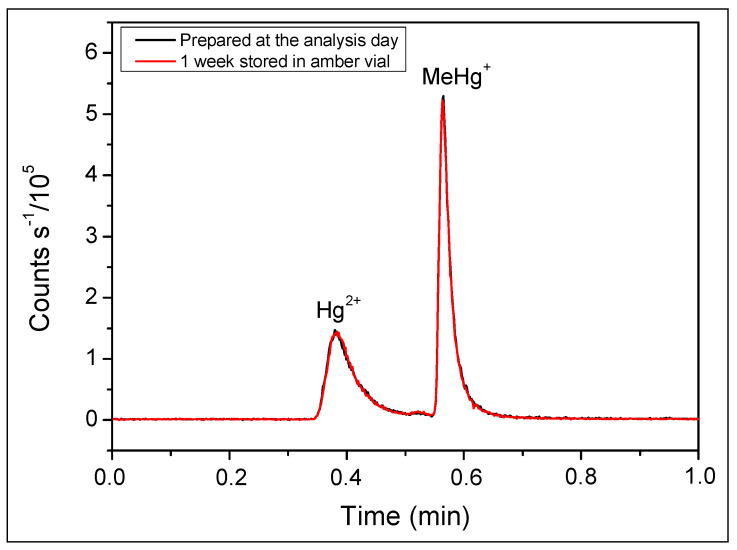
Chromatogram obtained by UHPLC-ICP-MS for 5 µL of solution containing Hg^2+^ and MeHg+ 10.0 µg L^−1^. Mobile phase: 0.05% *v/v* of 2-mercaptoethanol, 0.40% *m/v* of L-cysteine, 0.06 mol L^−1^ of ammonium acetate, and 5.0% *v/v* of methanol at 0.5 mL min^−1^. Stationary phase: column C18 4.6 mm × 150 mm, particle diameter 3.5 µm.

**Figure 4:**
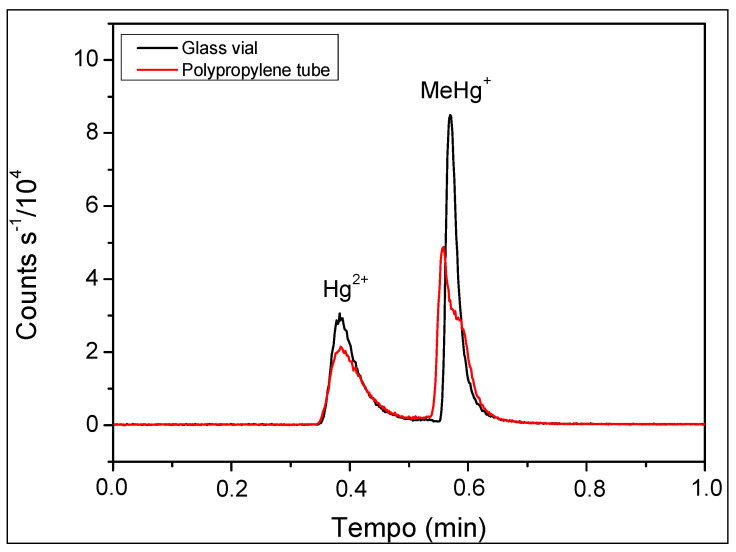
Chromatograms of 10.0 µg L^−1^ solutions of Hg^2+^ and MeHg^+^ stored in vial and in polypropylene tube for 72 h. Mobile phase: 0.05% *v v*^−1^ of 2-mercaptoethanol, 0.4% *w v*^−1^ of L-cysteine, 0.06 mol L^−1^ of ammonium acetate, and 5.0% *v v*^−1^ of methanol at 0.5 mL min^−1^. Stationary phase: column C18 2.1 mm × 50 mm, particle diameter 1.8 µm. Injection volume: 1.7 µL of solution.

The mercury species’ stabilities were confirmed through the sonication of standard solutions for up to 30 min, with no observed changes in chromatograms or concentrations (Table 2). A second test, performed by measuring the same solutions after a week stored in amber glass vials at approximately 25 °C, showed that the chromatograms overlapped (Figure 3), indicating the solution’s stability through the days. However, by comparing chromatograms of the same solution, fractioned and stored in a glass vial and a polypropylene tube at approximately 25 °C for 72 h, it was verified that the solution was unstable in the polypropylene tube once the analytical signal was reduced (Figure 4).

**Figure 5:**
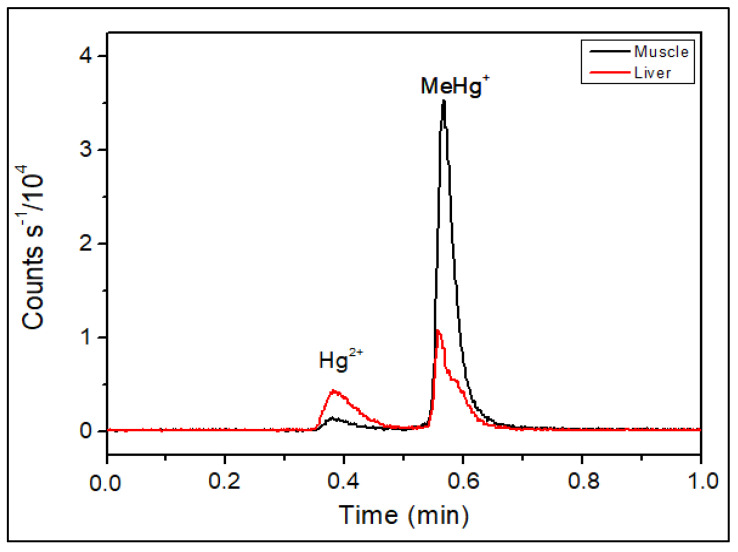
Chromatogram obtained by UHPLC-ICP-MS for muscle and liver fish extracts. Mobile phase: 0.05% *v v*^−1^ of 2-mercaptoethanol, 0.4% *w v*^−1^ of L-cysteine, 0.06 mol L^−1^ of ammonium acetate, and 5.0% *v v*^−1^ of methanol at 0.5 mL min^−1^. Stationary phase: column C18 2.1 mm × 50 mm, particle diameter 1.8 µm. Injection volume: 1.7 µL of solution.

Comparing the sum of Hg^2+^ and MeHg^+^ results obtained by UHPLC-ICP-MS with the value of total Hg determined by ICP-MS after acid digestion, it was determined that there was a recovery ranging from 92 to 105%. Considering the 95% confidence level, a statistical analysis using the Student’s *t*-test (Microsoft Excel, Redmond, WA, USA) confirmed the absence of a significant difference, showing evidence for the precision of our speciation method (Table 3 and Figure 5). Further research should explore the long-term stability of mercury species under various storage conditions, as variations of solutions in glass and polypropylene were observed (Figure 4) before extending the application of the method to other environmental matrices. Furthermore, the potential for the interconversion of mercury species during analysis warrants investigation to ensure the integrity of the speciation data. 

The results obtained until this moment are promising and demonstrate that the method has adequate selectivity, precision, and accuracy; however, the method will be adequately validated in the future.

## 4. Conclusions

The transition from HPLC-ICP-MS to UHPLC-ICP-MS for mercury speciation analysis marks a significant advancement in analytical efficiency. Our optimized method demonstrates a remarkable tenfold increase in speed, enabling the determination of Hg^2+^ and MeHg^+^ within a mere minute. This enhancement not only accelerates the analytical process but also substantially reduces the consumption of both the mobile phase and argon gas by factors of 11 and 5.5, respectively, contributing to a more sustainable analysis. In practical terms, this efficiency gain translates to an increase from 49 analyses to 270 analyses per argon cylinder, optimizing both time and financial resource use. These characteristics demonstrate that the UHPLC methodology was able to reduce the time and consequently the cost of analysis and reagent consumption, in addition to presenting an improvement in the selectivity and sensitivity of the analytes, proving the benefits of working in search of green methods. The use of UHPLC is a future trend, as small-diameter chromatographic columns coupled to ICP-MS also allow us to better obtain LoDs in addition to faster separations than with HPLC. The need to decrease LoDs is noticeable in the references due to the intensive search to minimize extra contributions from columns and dead volumes throughout the system. This is achieved through the optimization of system components, chromatographic columns with small particle sizes, and the minimization of tubing length and internal diameter.

Our investigation into the stability of mercury solutions has yielded reassuring results. Even after 30 min of sonication, we observed no volatilization or interconversion of species, affirming the reliability of our sample preparation method. Storage studies further corroborated the stability of solutions in glass vials for at least one week, a stability not mirrored in polypropylene tubes.

Accuracy testing using two different reference materials has validated the precision of our method. Given the complexity of the tested samples, we are confident in the method’s applicability to other matrices, such as environmental samples (soil, sediment, animal tissues), as well as samples that can be used in toxicological studies to assess exposure to mercury (blood, plasma, and hair). A faster, lower-cost, and more reliable method impacts positively on environmental and biological monitoring studies and routine labs due to the large number of samples that can be analyzed. The combination of versatility, demonstrated speed, and efficiency makes UHPLC-ICP-MS an outstanding tool for monitoring the environment and assessing the danger of mercury contamination.

## Figures and Tables

**Table 1:** ICP-MS operational conditions for speciation analysis.

Parameter	Condition/Value
RF power (KW)	1.6
Scan mode	Time-Resolved Analysis
Argon flow rate (L min^−1^)	
Plasma	15
Auxiliary	0.8~1.0
Carrier	1.07
Peristaltic pump speed (drain/waste)	0.10 rps
Nebulizer	Micro Mist
Spray chamber	Scott (double pass)
Torch injector diameter (mm)	2.5
Interface	Platinum cones
Sampler cone (mm)	0.90
Skimmer (mm)	0.45
Resolution	0.5 amu
Sample depth	8.0
Sweeps/replicate	100 sweeps
Integration time	0.1 s
Peak pattern	3 points
Isotope	^202^Hg

**Table 2:** Concentrations of Hg^2+^ and MeHg^+^ after sonication of 1.0, 5.0, and 10.0 µg L^−1^ aqueous standard solutions containing both species in presence of 2-mercaptoethanol (0.10%), L-cysteine (0.05%), and of HCl (0.10%). All solutions were in triplicate.

Species	Concentration (µg L^−1^)	Time (min)		
		0 min	15 min	30 min
Hg^2+^	1.0	0.89 ± 0.05	1.02 ± 0.04	1.03 ± 0.19
	5.0	5.31 ± 0.12	5.08 ± 0.13	5.34 ± 0.00
	10.0	10.58 ± 0.21	9.76 ± 0.19	10.29 ± 0.21
MeHg^+^	1.0	1.04 ± 0.01	1.02 ± 0.04	1.02 ± 0.03
	5.0	4.95 ± 0.20	5.04 ± 0.10	4.99 ± 0.09
	10.0	10.03 ± 0.13	10.04 ± 0.22	10.04 ± 0.15

**Table 3:** Concentrations of total Hg, Hg^2+,^ and MeHg^+^ in fish tissue samples.

Sample	Total Hg (ng g^−1^)	Hg^2+^(ng g^−1^)	MeHg^+^(ng g^−1^)	Sum of Hg Species Compared to Total Hg (%)
Muscle	607 ± 25	0	557 ± 75	92
Liver	322 ± 3	129 ± 18	208 ± 6	105

## Data Availability

Dataset available on request from the authors.
